# Reviewed article: Silva JRS, Arruda RG, Silva BO, Menezes ESB, Diniz CMM, Gomes JPA, Araújo BC, Rêgo MJBM, Pitta MGR, Pereira MC. High prevalence of respiratory diseases: a population-based ecological study, Sertão do Araripe, 2008-2019. Epidemiol Serv Saude. 2025:34;e20240519

**DOI:** 10.1590/S2237-96222025v34e20240519.a

**Published:** 2025-09-15

**Authors:** Osmar Cardoso

**Affiliations:** 1 Universidade Federal do Piauí, Teresina, PI, Brazil Universidade Federal do Piauí Teresina PI Brazil

## Peer review A

## Questionnaire

Does the manuscript contain new and significant information to justify publication? Yes

Does the Abstract (Summary) clearly and accurately describe the content of the article? Yes

Is the problem significant and concisely stated? Yes

Are the methods described comprehensively? Yes

Are the interpretations and conclusions justified by the results? Yes

Is adequate reference made to other work in the field? Yes

## Manuscript Structure

Length of article is: Adequate

Number of tables is: Adequate

Number of figures is: Adequate

Please state any conflict(s) of interest that you have in relation to the review of this paper. None

Interest: Excellent

Quality: Excellent

Originality: Excellent

Overall: Excellent

Recommendation: Minor Revision

## Comments

A pesquisa é importante pq contribui para o SUS ao fornecer dados sobre uma ativdade econômica importante, mas que gera adoecimento principalmente em crinças de 1 a 4 anos e idosos, evidenciando o impacto ambiental da atividade, mas também mostrando a efetividade dos serviços assistenciais do SUS local, uma vez que não ocorre o aumento da mortalidade por pneumonia.

Minhas preocupações com o artigo são a não explicação convincente do adoecimento especificamente da penumonia relacionado aos resíduos gesseiros e portanto solicito colocar maiores explicações sobre isso e pq outras patologias respiratórias não se manifestaram e sobre os mapas de georreferenciamento, entendo que precisam de legendas mais adequadas que localizem o leitor melhor de qual região é o foco do trabalho e o que deve ser levado em conta ao observar os mapas.

